# Anti-inflammatory activity and neutrophil reductions mediated by the JAK1/JAK3 inhibitor, CP-690,550, in rat adjuvant-induced arthritis

**DOI:** 10.1186/1476-9255-7-41

**Published:** 2010-08-11

**Authors:** Debra M Meyer, Michael I Jesson, Xiong Li, Mollisa M Elrick, Christie L Funckes-Shippy, James D Warner, Cindy J Gross, Martin E Dowty, Shashi K Ramaiah, Jeffrey L Hirsch, Matthew J Saabye, Jennifer L Barks, Nandini Kishore, Dale L Morris

**Affiliations:** 1 Worldwide Research, Pfizer Global Research & Development, Chesterfield, MO, USA; 2 Drug Safety R&D, Pfizer Global Research & Development, Chesterfield, MO, USA; 3 Pharmacokinetics, Dynamics and Metabolism, Pfizer Global Research & Development, Chesterfield, MO, USA

## Abstract

**Background:**

The Janus kinase (JAK) family of tyrosine kinases includes JAK1, JAK2, JAK3 and TYK2, and is required for signaling through Type I and Type II cytokine receptors. CP-690,550 is a potent and selective JAK inhibitor currently in clinical trials for rheumatoid arthritis (RA) and other autoimmune disease indications. In RA trials, dose-dependent decreases in neutrophil counts (PBNC) were observed with CP-690,550 treatment. These studies were undertaken to better understand the relationship between JAK selectivity and PBNC decreases observed with CP-690,550 treatment.

**Methods:**

Potency and selectivity of CP-690,550 for mouse, rat and human JAKs was evaluated in a panel of *in vitro *assays. The effect of CP-690,550 on granulopoiesis from progenitor cells was also assessed *in vitro *using colony forming assays. *In vivo *the potency of orally administered CP-690,550 on arthritis (paw edema), plasma cytokines, PBNC and bone marrow differentials were evaluated in the rat adjuvant-induced arthritis (AIA) model.

**Results:**

CP-690,550 potently inhibited signaling through JAK1 and JAK3 with 5-100 fold selectivity over JAK2 in cellular assays, despite inhibiting all four JAK isoforms with nM potency in *in vitro *enzyme assays. Dose-dependent inhibition of paw edema was observed *in vivo *with CP-690,550 treatment. Plasma cytokines (IL-6 and IL-17), PBNC, and bone marrow myeloid progenitor cells were elevated in the context of AIA disease. At efficacious exposures, CP-690,550 returned all of these parameters to pre-disease levels. The plasma concentration of CP-690,550 at efficacious doses was above the *in vitro *whole blood IC50 of JAK1 and JAK3 inhibition, but not that of JAK2.

**Conclusion:**

Results from this investigation suggest that CP-690,550 is a potent inhibitor of JAK1 and JAK3 with potentially reduced cellular potency for JAK2. In rat AIA, as in the case of human RA, PBNC were decreased at efficacious exposures of CP-690,550. Inflammatory end points were similarly reduced, as judged by attenuation of paw edema and cytokines IL-6 and IL-17. Plasma concentration at these exposures was consistent with inhibition of JAK1 and JAK3 but not JAK2. Decreases in PBNC following CP-690,550 treatment may thus be related to attenuation of inflammation and are likely not due to suppression of granulopoiesis through JAK2 inhibition.

## Background

CP-690,550, a selective inhibitor of the JAK family of protein tyrosine kinases, is being developed as an immunosuppressive and anti-inflammatory agent for the treatment and prevention of acute allograft rejection, RA, psoriasis and other immune mediated diseases [1-6].

In clinical trials, CP-690,550 administration resulted in a dose-related decrease in PBNCs in active RA patients [7,8] within 2 weeks of treatment, but not in psoriasis patients [9], renal allograft patients [10] or normal volunteers [11] for up to 14 and 28 days of treatment, respectively. In the RA trial [7,8], as observed in other RA studies [12], patients were found to have baseline PBNCs which were at or above the upper limit of the reference range for normal human subjects. Following treatment with CP-690,550 for 2 weeks, PBNCs in these patients were found to decrease to within the normal reference range, and showed a strong dose-related correlation with the anti-inflammatory activity of the compound [13].

Multiple inflammatory cytokine receptors signal through pathways involving JAK1 and JAK3, and their inhibition with CP-690,550 likely leads to anti-inflammatory and immunosuppressive activity. Conversely, JAK2 is required for signaling through several growth factor receptors and is important for myeloid and erythroid hematopoiesis [14-16].

The aim of the current study was to characterize the potency and selectivity of CP-690,550 for the JAK family members and to determine if PBNC reductions in the context of arthritis are related to the anti-inflammatory efficacy of CP-690,550 (through JAK 1 and JAK3 inhibition), or due to inhibition of hematopoiesis through inhibition of JAK2 at efficacious exposures. The *in vitro *potency and selectivity of CP-690,550 were determined using recombinant human kinases and whole blood cytokine induced STAT phosphorylation assays. In studies published previously, CP-690,550 was shown to inhibit arthritis development and bone destruction in the rat AIA model [17]. In the current study, rat AIA was used to characterize the *in vivo *effects of inflammation and CP-690,550 treatment on PBNCs and bone marrow myeloid progenitors, and *in vitro *bone marrow progenitor cell differentiation assays were used to determine the direct effects of CP-690,550 on granulopoiesis. Additionally, pharmacokinetic and pharmacodynamic modeling was used to determine the drug concentration-effect relationship between JAK kinase inhibition and neutrophil reductions in the non-clinical and clinical studies.

Results of this study suggest that the CP-690,550 mediated reduction in PBNCs in the rat AIA model, and likely in human RA patients, is due to the anti-inflammatory action of the compound by the suppression of cytokines and chemotactic factors which elevate neutrophil counts in the peripheral compartment.

## Methods

### Enzyme Potency and Selectivity Assays

Recombinant kinase domains of JAK2 and JAK3 were purchased from Invitrogen (Madison, WI), and recombinant GST-fusions of JAK1 (residues 852-1142) and TYK2 (residues 870-1187, containing a C1187 S modification) kinase domains were expressed and purified at Pfizer Laboratories. MgATP was obtained from Sigma Chemical Company (St. Louis, MO). JAKtide (FITC-KGGEEEEYFELVKK) and IRS-1 (5-FAM-KKSRGDYMTMQIG) peptides were purchased from American Peptide Company (Sunnyvale, CA). FITC-conjugated antibodies against human CD8, mouse CD8, mouse CD11b and rat CD3; PE-conjugated antibodies against human CD3, human CD14, mouse CD3 and rat CD4; and AlexaFluor^®^647 (Ax647) conjugated pSTAT1, pSTAT3, pSTAT5 and pSTAT6 monoclonal antibodies were from BD Biosciences (San Jose, CA). The PE-conjugated antibody against mouse F4/80 was from eBioscience (San Diego, CA). Recombinant human, mouse and rat cytokines were from R&D Systems (Minneapolis, MN).

### *In Vitro *Kinase Assays

A peptide mobility shift assay was used to quantify the phosphorylation of JAKtide (for JAK2 and JAK3) or IRS-1 (for JAK1 and TYK2) peptide substrates. Endpoint reactions were carried out at the apparent K_m (MgATP) _for each enzyme (4 μM for JAK2 and JAK3, 40 μM for JAK1 and 7 μM for TYK2) in the presence of CP-690,550 and 1 μM substrate. Samples were analyzed by LabChip 3000 from Caliper Life Sciences (Hopkinton, MA). Data was analyzed using HTS Well Analyzer Software from Caliper Life Sciences to determine the amount of product formed, and was expressed as percent of control activity based on uninhibited and no enzyme controls. Dose-response data were fit using 4 parameter logistic fit software to determine IC_50_. To determine the inhibition constant (K_i_) for each enzyme, initial velocities were measured at varying concentrations of CP-690,550 and MgATP. The data from each experiment were fit to competitive, noncompetitive and uncompetitive inhibition models, to determine nonlinear fit and Lineweaver-Burk transformations [18].

### *In Vitro *Whole Blood STAT Phosphorylation Assays

Heparinized normal human, DBA/1 mouse or Lewis rat whole blood was pre-incubated with CP-690,550 for 1 hour and stained with lineage-specific antibodies. Specifically, species-specific antibodies to CD3 and CD8 were used to identify human, mouse and rat total T cells or the CD8 subset, while anti-CD14 was used to label human monocytes, anti-CD11b and anti-F4/80 were used to label mouse monocytes, and anti-CD3 and anti-CD4 were used to identify rat monocytes (CD3^-^, CD4^+^). Blood was stimulated with or without cytokine (IL-2, IL-4, IL-6, IL-7, IL-15, IL-21 and IFN-γ at 100 ng/mL, GM-CSF at 20 ng/mL or IFN-α at 1000 units/mL) at 37°C for 8-20 minutes, and activation was stopped by the addition of Lyse/Fix Buffer (BD Biosciences) following the manufacturer's protocol. Cells were washed, permeabilized in ice-cold Perm Buffer III (BD Biosciences) for 20 minutes, and stained with Ax647-conjugated phospho STAT-specific monoclonal antibodies. Flow cytometric analysis (FACS) was performed on a FACSCalibur equipped with an HTS plate loader (BD Biosciences) running Cellquest software. Twenty thousand cellular events were collected per sample, and listmode data was analyzed using FlowJo software (Treestar, Ashland, OR). Ax647 geometric mean channel fluorescence (MCF) was determined from either the CD3^+ ^T cell population, the CD3^+^/CD8^+ ^T cell subset, or from CD14^+ ^human monocytes, CD11b^+^/F4/80^+ ^mouse monocytes or CD3^-^/CD4^+ ^rat monocytes. Percent of control STAT phosphorylation was determined at each concentration of CP-690,550 by comparison of Ax647 MCF with those from unstimulated and cytokine stimulated controls. IC_50 _values were determined using four parameter logistic fit software.

### Colony Forming Cell Assays

Colony forming cell (CFC) assays were performed by StemCell Technologies, Inc. as previously described by Pereira [19]. CP-690,550 was prepared in DMSO and added to culture dishes at a final concentration of 0.1% DMSO. Human bone marrow hematopoietic cells (Lonza, Walkersville, MD), were added to the appropriate media formulations to obtain final plating concentrations. CFC assays contained 30% fetal bovine serum/1% bovine serum albumin. Cultures were plated and incubated at 37°C, 5% CO_2, _and colonies were evaluated and scored microscopically. A dose-response graph was generated plotting the log of the compound concentration versus the percentage of control colony growth using Microcal Origin software. IC_50 _values were calculated using the Boltzman equation. CP-690,550 was evaluated in two separate experiments and each IC_50 _value in Table 1 is a representation of 6 separate dose-response curves. Cytotoxicity was evaluated in Huh 7 cells using the MTT (3-(4,5-dimethylthiazol-2-yl)-2 diphenyltetrazolium bromide; Sigma) reduction assay.

**Table 1:** CP-690,550 *In Vitro *Potency, Selectivity and Inhibition of Myeloid Progenitor Cell

**Differentiation CP-690,550 Kinase Inhibition**^**A**^						
**Recombinant human kinase**		**IC**_**50 **_**(nM)**			**K**_**i **_**(nM)**	

JAK1		3.2 ± 1.4			0.68 ± 0.12	
JAK2		4.1 ± 0.4			0.99 ± 0.04	
JAK3		1.6 ± 0.2			0.24 ± 0.02	
TYK2		34.0 ± 6.0			4.39 ± 0.27	

**CP-690,550 Inhibition of Cytokine Signaling in Human Whole Blood**^**A**^						

**Cytokine**	**JAK**	**pSTAT**	**CD3**^**+ **^**T cells** **IC**_**50 **_**(nM)**		**Monocytes** **IC**_**50 **_**(nM)**	

IL-2	1/3	5	28 ± 5		NA	
IL-4	1/3	6	50 ± 5		NA	
IL-7	1/3	5	38 ± 9		NA	
IL-6	1/2	1	54 ± 7		NA	
IL-6	1/2	3	367 ± 49		406 ± 68	
IFN-α	1/TYK2	1	44 ± 4		148 ± 41	
IFN-γ	1/2	1	NA		178 ± 38	

**Species Comparison of CP-690,550 Inhibition of Cytokine Signaling in Whole Blood**^**A**^						

**Cytokine**	**JAK**	**pSTAT**	**Cells**	**Human** **IC**_**50 **_**(nM)**	**Mouse** **IC**_**50 **_**(nM)**	**Rat** **IC**_**50 **_**(nM)**

IL-15	1/3	5	CD8^+ ^T cells	56 ± 6	42 ± 12	NA
IL-21	1/3	3	CD3^+ ^T cells	25 ± 6	NA	187 ± 48
IL-6	1/2	1	CD3^+ ^T cells	54 ± 7	185 ± 46	62 ± 14
GM-CSF	2	5	Monocytes	1377 ± 185	4379 ± 655	877 ± 171

**CP-690,550 Inhibition of Human and Mouse Myeloid Progenitor Cell Differentiation**^**B**^						

**Total Human Myeloid Colonies** **IC**_**50 **_**(nM)**	**Human** **CFU-G**^**C **^**Colonies** **IC**_**50 **_**(nM)**		**Total Mouse Myeloid Colonies** **IC**_**50 **_**(nM)**		**Mouse** **CFU-G Colonies** **IC**_**50 **_**(nM)**	

870 ± 120	930 ± 30		1210 ± 490		1128 ± 524	

^A ^Dose-response data were fit using four parameter logistic equations to determine IC50, and represent the mean ± SEM from at least three independent studies. Inhibition constants (Ki) were determined by measuring initial velocities for each enzyme in the presence of varying MgATP and inhibitor concentrations. NA (not applicable), signaling through the indicated cytokines was insufficient to determine inhibition. ^B ^IC_50 _values were derived from the Boltzman Fit Model and represent the mean ± (standard deviation, SD); ^C ^CFU-G = colony forming units - granulocytes.

### Rat Adjuvant-Induced Arthritis

Female Lewis rats, 160-180 grams, were obtained from Harlan Laboratories (Indianapolis, IN). Food and water were available ad libitum. The Pfizer Institutional Animal Care and Use Committee reviewed and approved the animal use in these studies. The animal care and use program is fully accredited by the Association for Assessment and Accreditation of Laboratory Animal Care. Adjuvant was prepared with *Mycobacteria butyricum *(Lot #264010, Difco Laboratories, Detroit, MI) in a 15 mg/mL suspension with squalene oil (Sigma) with a PT3100 homogenizer (Kinematica, Bohemia, NY). On day 0, rats were anesthetized with CO_2_/O_2 _and three 50 μL injections were administered subcutaneously at the base of the tail. Rats were dosed orally with either vehicle (0.5% methylcellulose/0.025% Tween-20) alone or CP-690,550 suspended in vehicle. Dosing was initiated on day 14 and continued twice daily through day 21 post-immunization. The volume of both the right and left hind paw of each rat was measured by plethysmometer (Model 7140, Ugo Basile, Collegeville, PA) and the combined volume of both paws was calculated. Blood was collected in EDTA microtainer tubes (Becton Dickinson, Franklin Lakes, NJ) under CO_2_/O_2 _anesthesia. Absolute total neutrophil counts in each sample were measured from whole blood using a Cell-Dyne 3700 instrument (Abbott Laboratories, Abbott Park, IL). Plasma CP-690,550 concentrations were measured using LC/MS/MS techniques. Population plasma levels for each dose were assessed based on one-compartment pharmacokinetics and mean elimination rate estimates. Daily (24 hr) area-under-the-curve (AUC) exposures were subsequently calculated based on the trapezoidal rule. Pharmacodynamic values were assessed on day 21 by calculating the mean paw volume change for each treatment group compared to the mean vehicle control. These values were normalized with the mean vehicle control paw volume change to yield a fractional pharmacodynamic response. The mean fractional pharmacodynamic response values were plotted against their mean pharmacokinetic AUC values and modeled with an Emax non-linear regression model with a maximal pharmacodynamic response constraint of 1 (GraphPad Prism^® ^v. 5.01; GraphPad Software Inc., La Jolla, CA).

### Rat Bone Marrow Analysis by Flow Cytometry

Rat bone marrow suspensions were collected, prepared and analyzed as described by Criswell, et al [20]. Total nucleated cell count (TNCC) was determined using a hematology analyzer (Advia 2120, Siemens, Terrytown, NY). FACS was performed on a Beckman Coulter FC500 equipped with a 15-mW argon-ion laser set at 488 nm and calibrated prior to use. Fluorescent signals were obtained through bandpass filters at 525 nm for the green fluorescence of DCF analysis and at 575 nm for the red fluorescence of PE-tagged lymphocyte analysis. A minimum of 35,000 nucleated cells for DCF analysis and 70,000 nucleated cells for lymphocyte analysis were acquired on each bone marrow sample with listmode date files storing values for forward scatter (FS), FL1, and log integral red fluorescence (FL2). CXP software was used for data analysis of the following bone marrow subpopulations: maturing and proliferating myeloid, maturing and proliferating erythroid, megakaryocytes and lymphocytes. Summary statistics (mean and standard deviation) were calculated for each treatment group for percentage and absolute values, as well as, myeloid:erythroid (M:E) ratios. Student's t-tests were performed to assess statistical differences.

### Plasma Cytokine Immunoassays

Cytokines were detected in plasma using Luminex-based rat IL-17 LINCOplex kits (Millipore/LINCO, St. Charles, MO) and Luminex 200 instrumentation (Luminex Corporation, Austin, TX) following the manufacturer's instructions, or Meso Scale Discovery Ultra -Sensitive rat IL-6 kits and Sector Imager 6000 instrumentation (Meso Scale Discovery, Gaithersburg, MD) following the manufacturer's protocol.

## Results

### Enzyme Inhibitory Potency and Selectivity

The potency of CP-690,550 against JAK1, JAK2, JAK3 and TYK2 enzymes was measured using recombinant human kinase domains, together with Caliper microfluidic technology. Inhibition activity was evaluated at fixed concentrations of MgATP corresponding to the apparent K_m (MgATP) _(refer to *Methods *section for appropriate peptide substrate used for each enzyme), and is summarized in Table 1. CP-690,550 demonstrated nM potency against all JAK enzymes, with the rank order of potency being JAK3 > JAK1, JAK2 > TYK2. To elucidate the mechanism of inhibition, we collected initial velocities for each enzyme at varying concentrations of CP-690,550 and MgATP. The data from each experiment were fit to competitive, noncompetitive and uncompetitive inhibition models, and in each case the pattern of inhibition could best be described by a competitive model. These results suggest that for each JAK family member, CP-690,550 competes with ATP for binding to the active site of the kinase domain. Nonlinear fit and Lineweaver-Burk transformations were used to calculate the K_i _for each enzyme as shown in Table 1. K_i _values for the JAKs showed similar rank order potency to the IC_50 _values.

### STAT Phosphorylation in Whole Blood Leukocytes

Since JAKs directly phosphorylate STAT proteins in response to specific cytokine stimulation, measuring the extent of STAT phosphorylation in cells is an indirect measurement of JAK inhibition. The potency and selectivity of CP-690,550 were therefore assessed *in vitro *in whole blood using intracellular flow cytometry to measure STAT phosphorylation. Normal human, mouse or rat whole blood pre-treated with CP-690,550 was stimulated with cytokines, and STAT phosphorylation downstream of receptor-associated JAK activation was monitored in monocyte and T cell populations as summarized in Table 1. Multiple cytokine activated JAK/STAT signaling pathways were potently inhibited with IC_50 _values below 200 nM. In human T cells these pathways included γc-cytokine signaling through JAK1/3, as well as, IL-6 and IFN-α activation of STAT1 through JAK1/2 or JAK1/Tyk2, respectively. In contrast, we observed a 7-fold reduction in potency against IL-6 driven STAT3 phosphorylation compared to activation of STAT1 by the same cytokine. In monocytes, comparable inhibition of IFN-α and IFN-γ signaling was observed even though potency was 3-4-fold reduced compared to T cells. Additionally, the inhibitor demonstrated reduced potency against IL-6 activation of STAT3 and significantly reduced inhibition of JAK2-mediated GM-CSF signaling. Given the caveat that potency assessments against the various JAK/STAT pathways were made in differing cell types, these data suggested that CP-690,550 might have greater selectivity for JAK1 and JAK3 signaling pathways in T cells despite comparable activity against all JAK family members in isolated kinase assays. The selectivity of CP-690,550 was also assessed in mouse and rat whole blood (Table 1), with results similar to those observed in human assays. Cytokine signaling through JAK1 and JAK3 pathways in T cells appeared to be more potently inhibited than did JAK2 signaling in monocytes.

### Colony Forming Cell Assay

CP-690,550 was evaluated in the CFC and MTT cytotoxicity assays (Table 1). Data are presented for both total myeloid colonies, as well as, for colony forming units-granulocytes (CFU-G). The IC_50 _values for CP-690,550 inhibition of human total myeloid and CFU-G colony formation were 0.87 and 0.93 μM, respectively.

The concentrations at which CP-690,550 is shown to have an effect on hematopoietic stem cell colony formation and differentiation *in vitro *correlated well with human *in vitro *selectivity data and JAK 2 inhibition (Table 1, human whole blood cytokine signaling assays). Free fraction compound concentrations for *in vitro *CFC assays were determined to be 0.67 ± 0.05 μM (n = 3) at a total *in vitro *concentration of 1 μM. This is consistent with free fraction compound concentrations in mouse and human plasma (mean of 0.67 and 0.61 respectively for concentration range of 0.5 to 8 μM). Therefore, free fraction compound concentrations were determined to be comparable between the CFC assays, *in vitro *whole blood assays, and *in vivo *pharmacology studies. Moreover, CP-690,550 was not found to be cytotoxic in Huh 7 hepatoma cells at concentrations up to 100 μM (data not shown), suggesting that the observed inhibition of myeloid colony formation is due to a specific effect of CP-690,550 on cell proliferation and differentiation.

### Rat AIA Model Characterization

**Figure 1:**
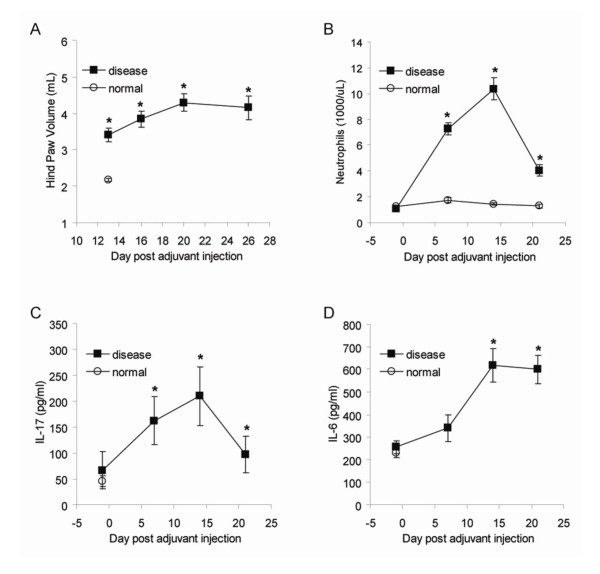
**Peripheral blood neutrophil count and the inflammatory cytokines IL-6 and IL-17 increase with disease in the rat AIA model**. Normal and AIA rats (n = 12 rats per group per timepoint) were characterized temporally post-adjuvant immunization. Hind paw volume was measured by volume displacement as an indication of joint arthritis, days 13 to 26 (A). Peripheral blood neutrophils were quantitated using a Cell-Dyne 3700 analyzer, days -1 to 21 (B). IL-17 (C) and IL-6 (D) were quantitated using immunoassays, days -1 to 21. (*) indicates statistical significance p ≤ 0.04 compared to normal controls. Data represented as the mean ± SEM.

Rats were immunized on day 0 and the development of arthritis, as indicated by increasing paw volume, was measured by volume displacement. As shown in Figure 1A, there was a significant increase in paw volume on day 13 post-immunization as compared to normal non-immunized controls. Elevations in paw volume peaked in the AIA rats on day 21 and remained elevated through day 28. Blood samples were collected for PBNC prior to immunization, and weekly through day 21 post-immunization. PBNC in AIA rats significantly increased compared to normal control animals on day 7, peaked on day 14, and declined on day 21 (Figure 1B). The inflammatory cytokines IL-17 and IL-6 were measured in plasma at the same time points as PBNC, and both proteins significantly increased with AIA disease progression (Figures 1C and 1D). IL-17 significantly increased on day 7, peaked on day 14, and decreased on day 21 showing the same temporal response to adjuvant immunization as PBNC. IL-6 increased in the AIA rats with levels peaking on day 14, but remained elevated through day 21, similar to the arthritic response.

### Characterization of CP-690,550 Effects in the Rat AIA model

**Figure 2:**
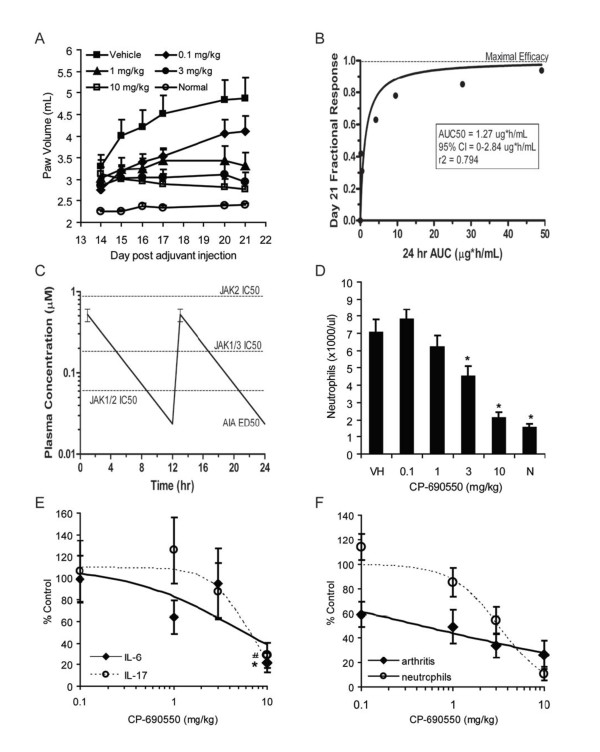
**CP-690,550 dose-dependently inhibits hind paw edema, PBNC, and serum inflammatory cytokine levels in the rat AIA model**. AIA rats were treated orally with CP-690,550 (n = 12 rats per treatment group), twice daily, starting on day 14 and continuing through day 21 post-adjuvant immunization. Arthritis was monitored by measuring hind paw volume using volume displacement (A). (*) indicates statistical significance p < 0.014 compared to vehicle control treated disease animals. Drug exposure-paw volume response relationship was determined (B). CP-690,550 ED_50 _plasma exposures were compared to *in vitro *rat whole blood IC_50 _potencies for JAK1/2, JAK2, and JAK1/3 (C). Whole blood was analyzed for PBNC using a Cell-Dyne analyzer on day 21 post immunization (D). (*) indicates statistical significance p < 0.002 compared to vehicle control treated disease animals. IL-17 and IL-6 were quantitated using immunoassays (E). IL-17 (#) indicates statistical significance p ≤ 0.059 and IL-6 (*) indicates statistical significance p ≤ 0.013 compared to disease vehicle control. Day 21 dose-related correlation between reductions in hind paw edema and PBNC in AIA rats (F). Data represented as the mean ± SEM (A-D) or the mean percent control ± SEM (E and F).

Milici et al. previously demonstrated that CP-690,550 treatment delivered via sub-cutaneous pumps dose-dependently inhibits arthritis development in the rat AIA model both by measuring hind paw volume and histological evaluation of inflammation and bone damage [17]. In the present study, CP-690,550 or vehicle control was administered orally to rats twice daily starting on day 14 when disease was clearly evident by increased paw volume, and was continued through day 21. As shown in Figure 2A, CP-690,550 treatment resulted in a dose-dependent inhibition of arthritis as indicated by decreased paw volume compared to vehicle treated control rats. Notably, CP-690,550 at a 10 mg/kg dose reduced paw volume to within the normal range by day 21. The drug exposure-paw volume response relationship is shown in Figure 2B, and represents an AUC_50 _of 4968 nM*hr or an ED_50 _of 0.55 mg/kg. In Figure 2C, the ED_50 _exposure is compared to the *in vitro *rat whole blood JAK1/2, JAK2, and JAK1/3 IC_50 _values of CP-690,550. These data suggest the importance of JAK1 and JAK3 inhibition for efficacy, since ED_50 _exposures were below those needed for JAK2 inhibition in whole blood.

PBNC were determined on day 21 post-immunization. As shown in Figure 2D, vehicle control treated AIA rats had a mean blood concentration of 7.0 × 10^3 ^neutrophils per μL compared to 1.5 × 10^3 ^per μL in normal vehicle treated rats, an approximately 4.5-fold increase with disease. CP-690,550 treatment dose-dependently decreased the disease-elevated neutrophil count to normal range at the 10 mg/kg dose. In contrast, decreases in PBNC were not observed in normal rats at dose levels up to 100 mg/kg (once per day) for up to 6 months in duration, although slight reductions in red blood cell parameters were observed at the 100 mg/kg dose level (data not shown). Furthermore, reductions in PBNC were not observed in non-human primates following exposures of up to 9 months duration [21]. CP-690,550 treatment dose-dependently decreased both IL-17 and IL-6 showing an approximately 80% inhibition of the AIA-induced increase compared to control levels (Figures 2E). CP-690,550 inhibited arthritis approximately 5 times more potently as compared to inhibition of PBNC (Figure 2F).

### Characterization of Bone Marrow Myeloid Precursors in AIA Rats

**Figure 3:**
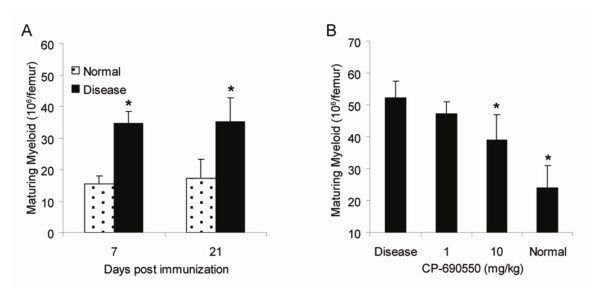
**CP-690,550 inhibits the increase in bone marrow myeloid precursors in the rat AIA model**. Bone marrow from normal and AIA rats (n = 12 rats per group) was analyzed for maturing myeloid precursors by flow cytometry at day 7 and day 21 post-adjuvant immunization (A). (*) indicates statistical significance p ≤ 0.0008 compared to normal animals. AIA rats were treated with CP-690,550 or vehicle control orally, once daily, days 14 to 21, and bone marrow was collected and analyzed on day 21 for maturing myeloid cells by flow cytometry (B). (*) indicates statistical significance p ≤ 0.05 compared to the disease vehicle control. Data represented as the mean ± SEM.

Bone marrow was harvested from normal (n = 12) and diseased (n = 12) rats on days 7 and 21 post immunization and the number of maturing myeloid cells was quantified by FACS. As shown in Figure 3A, the maturing myeloid cell number in AIA rat bone marrow was significantly increased compared to normal controls at both 7 and 21 days post immunization. On day 21, AIA rats had 35.2 × 10^6 ^cells per femur compared to 17.3 × 10^6 ^cells per femur in the normal rats, an approximately 2-fold increase with disease. Consistent with increased marrow myeloid cells, increases in TNCC and M:E ratio were also observed. AIA rats were treated with 1 and 10 mg/kg CP-690,550 or vehicle control (n = 12 per group) starting on day 14 post-immunization, and continued through day 21. On day 21, bone marrow was harvested from treated and normal rats and the myeloid precursors were quantitated. As shown in Figure 3B, CP-690,550 significantly inhibited the AIA-induced increase in maturing myeloid cells by approximately 50% at the 10 mg/kg dose. The decline in the marrow maturing myeloid population appears to be consistent with the restoration of PBNC to normal range following CP-690,550 treatment.

### Clinical Pharmacokinetics and Relationship to Neutrophil Count Reductions

The clinical pharmacokinetics of CP-690,550 showed good dose proportionality in various subject populations [10,11]. Collective non-compartmental pharmacokinetic modeling of available clinical data is indicated in Table 2. At doses from 5 to 10 mg BID, JAK2 IC_50 _(GM-CSF dependent pSTAT5 in whole blood) coverage is insignificant in comparison to JAK1/3 IC_50 _coverage (IL-21 dependent pSTAT3 in whole blood). However, JAK2 inhibition may increase at higher doses depending on the *in vitro *potency value used (Table 2). Doses as low as 5 mg BID are well-tolerated and efficacious in moderate to severe RA [13], suggesting the importance of JAK1 and JAK3 target coverage in the absence of JAK2 inhibition. Doses of 5 and 10 mg BID are currently being explored in Phase 3 clinical trials in RA (ClinicalTrials.gov identifier: NCT00847613). Therefore, exposures of CP-690,550 which are associated with efficacy and PBNC reductions in human RA patients and in the rat AIA model correlate primarily with the inhibition of JAK1 and JAK3 and not that of JAK2, based on the assay results in Table 1.

**Table 2:** Modeled Human Pharmacokinetic Parameters and JAK1/3 and JAK2 IC_50 _Coverage for CP-690,550

BID Dose mg	Cmax (nM)	24 hr AUC (nM*h)	**JAK1/3 IC**_**50 **_**Coverage Every** **12 hrs**^**A**^ **(range**^**B**^**)**	**JAK2 IC**_**50 **_**Coverage Every** **12 hrs**^**C, D**^ (range)
5	167	1548	7.8 hrs (7.0-8.8)	0 hrs^C ^(0-0) 0 hrs^D^
10	337	3097	10.3 hrs (9.6-11.3)	0 hrs (0-0) 0.3 hrs
15	503	4645	11.7 hrs (10.9-12.0)	0 hrs (0-0) 2.1 hrs
30	1006	9291	12.0 hrs (12.0-12.0)	0 hrs (0-0) 5.0 hrs

^A ^JAK1/3 human whole blood IC_50 _(IL-21 dependent pSTAT3) = 25 ± 6 nM; ^B ^range based on the upper and lower error around the IC_50 _where available; ^C ^JAK2 human whole blood IC_50 _(GM-CSF dependent pSTAT5) = 1377 ± 185 nM; ^D ^GM-CSF stimulated myelomonocytic HUO3 cell JAK2 IC_50 _= 324 nM [1].

## Discussion

In a recent analysis of kinase inhibitor selectivity within the human kinome, CP-690,550 was found to be highly selective for the JAK family kinases, and to have similar binding affinities for both JAK2 and JAK3 [22]. While binding to the kinase domain of JAK1 was not examined in that study, it has been suggested that therapeutic efficacy of CP-690,550 in some indications may be due to cross-over inhibition of other JAKs [23]. As we have demonstrated in the present study, CP-690,550 is a potent inhibitor of all JAKs in *in vitro *kinase assays where truncated kinase domain constructs were used. The K_i _and IC_50 _were measured for each enzyme, and the relative JAK enzyme rank order potency for CP-690,550 is comparable using either measure of inhibitory potency.

Inhibition of JAK-dependent STAT phosphorylation in whole blood was also examined as a measure of JAK potency and selectivity. The comparable potency observed in human T cells stimulated with several γc cytokines suggests that CP-690,550 is capable of inhibiting multiple STAT pathways equally (IC_50 _25-56 nM) within the same cell population. Similar potency was also observed against IL-6 and IFN-α driven STAT1 phosphorylation in T cells, whereas IL-6 activation of STAT3 was less sensitive. Although the gp130 component of the IL-6 receptor complex can associate with JAK1, JAK2 or TYK2, JAK1 plays an essential role in cell signaling, since JAK1-deficient cells fail to respond to IL-6 or other gp130 cytokines [24]. Thus, while CP-690,550 can inhibit cytokine activation pathways associated with JAK1, subtle differences in JAK or STAT utilization by specific receptors may influence inhibitor potency and selectivity. Further examination of the mechanism behind differential JAK inhibitor sensitivity of IL-6 signaling pathways in T cells will be intriguing. In monocytes, CP-690,550 had reduced activity compared to T cells, yet both type I and type II interferon signaling was inhibited with IC_50 _values below 200 nM, while GM-CSF signaling through a receptor that only utilizes JAK2 was significantly less affected. It is unclear mechanistically how CP-690,550 might spare JAK2 in cellular assays, although one explanation for reduced cellular potency could be that a cytoplasmic activator of JAK2, such as SH2-Bβ, can partially counteract the effects of kinase inhibition [25]. Other possible explanations could be JAK-specific differences in inhibitor potency between isolated kinase domains and full-length enzymes or even against JAK hetero/homodimers. The selectivity profile of CP-690,550 in mouse and rat whole blood was similar to that observed in human, suggesting that in contrast to its activity on isolated JAK kinases, CP-690,550 may have selectivity for JAK1 and JAK3 over JAK2 in whole blood or in cellular assays where JAKs are in their native conformation. If JAK2 sensitivity to CP-690,550 is indeed reduced in cells, our findings suggest that inhibition of the enzyme may not be required to block signaling through receptors for IL-6 and IFN-γ, as inhibition of JAK1 could be sufficient.

The two most likely mechanistic hypotheses which could explain observed clinical reductions in PBNC in RA subjects by CP-690,550 are: 1) suppression of bone marrow myeloid progenitor cell differentiation via a loss of selectivity and inhibition of JAK2; and 2) suppression of chemotactic and hematopoietic growth factors as an extension of the anti-inflammatory activity of CP-690,550.

Several lines of evidence suggest that the first hypothesis is not valid at fully efficacious doses of CP-690,550. First, reductions in PBNC were not observed non-clinically (in rodents (data not shown) and non-human primates [21] for up to 6 and 9 months of treatment, respectively; although a slight reduction in red blood cell parameters was observed in both species at dose levels where inhibition of JAK2 would be expected (data not shown)) or clinically in healthy volunteers, psoriasis and allograft patients (for up to 14 and 28 days of treatment, respectively) [9,10,21]. Secondly, RA patients in general, and most patients enrolled in the CP-690,550 RA clinical trial, were found to have elevated baseline PBNC, and treatment with CP-690,550 resulted in reductions in PBNC to within the normal human reference range [7,8]. Thirdly, *in vitro *human selectivity data suggests that clinically efficacious doses of CP-690,550 of up to 30 mg would inhibit the JAK1 and JAK3 enzymes, but not JAK2 (Tables 1 and 2).

It has previously been demonstrated that JAK2 is important in the signal transduction cascade for several hematopoietic growth factor receptors, including granulocyte-colony stimulating factor (G-CSF) and granulocyte macrophage-colony stimulating factor (GM-CSF) [3,14-16]. It is this established role of JAK2 in regulating granulocyte hematopoiesis that suggests the involvement of JAK2 inhibition in the PBNC reductions in RA patients.

Milici et al previously showed that CP-690,550 treatment in the rat AIA model inhibited arthritis development and bone destruction [17]. In the present study, we used the rat AIA model and an *in vitro *model of human and rodent (mouse) myeloid progenitor cell differentiation to characterize the effects on PBNC observed in RA patients. Additionally, we used pharmacokinetic and pharmacodynamic (PK/PD) modeling to determine the exposure-effect relationships and correlations between JAK inhibition, efficacy and PBNC reductions in both the non-clinical models and in RA patient populations.

Results from this study demonstrate a strong PK/PD relationship between inhibition of JAK1 and JAK3, efficacy, and the inhibition of inflammatory cytokines and neutrophilia in the rat AIA model with CP-690,550 (Tables 1 and 2, Figures 1 and 2). Increases in arthritis, inflammatory cytokines and PBNC in AIA rats correspond to increasing disease severity and progression. CP-690,550 dose-dependently inhibits all three parameters to levels observed in normal animals. Additionally, inhibition of arthritis occurs at a lower dose than the reductions in PBNC (Figure 2F), further suggesting that the reductions in neutrophils are secondary to inhibition of inflammation. Neither generalized myelosuppression nor neutropenia were observed in the rat AIA model, or in normal animals [21], even at exaggerated clinically efficacious exposures of CP-690,550.

Neutrophilia observed in the rat AIA model was also determined to be, at least in part, due to an increase in granulopoiesis in the bone marrow, and this effect was inhibited at a fully efficacious dose of CP-690,550 (Figure 3). These effects correlated with inhibition of both IL-17 and IL-6 which, in addition to TNF-α, IL-8 and GM-CSF, are pivotal inflammatory cytokines linked to the inflammation, neutrophilia and neutrophil chemotaxis that promote the progression of arthritic disease [26-30].

In *in vitro *hematopoiesis assays, CP-690,550 had no effect on human myeloid progenitor cell differentiation at concentrations which fully inhibit cytokine signaling pathways through JAK1 and JAK3 but do not appear to inhibit JAK2 in whole blood (Table 1), and are fully efficacious in rodent arthritis models and in human RA patients (Table 2, Figure 2) [17]. At exaggerated *in vitro *concentrations which exceed selectivity against JAK1 and JAK3 and do inhibit cytokine signaling through JAK2, inhibition of myeloid progenitor cell differentiation was observed (Table 1). Similar results were observed in the mouse, although the IC_50 _values were reduced relative to human (Table 1). These results are consistent with previous reports demonstrating a role of JAK2 in G-CSF and GM-CSF signaling and myeloid progenitor cell differentiation [14-16,26]. Although we did not directly evaluate inhibition of JAK2 or *in vitro *granulopoiesis in the rat, inhibition of arthritis, cytokines and neutrophilia in the AIA model were observed at plasma exposures that were well below the human JAK2 IC_50 _and effects on GM-CSF stimulated STAT phosphorylation in the rat whole blood assay. Furthermore, we did not observe any inhibitory effects on erythropoiesis in the rat (normal or AIA) over the course of CP-690,550 treatment, which would have been expected if JAK2 was inhibited [14,16]. However due to the extended half-life of RBCs such analysis is limited in this model. These findings were consistent with those of Manshouri et al, who demonstrated minimal inhibition of human ex-vivo expanded erythroid progenitors at CP-690,550 concentrations up to 1 μM [31]. Similarly, in a recent publication by Lin et al., dose-dependent inhibition of erythropoietin-driven reticulocyte formation by CP-690,550 in mice was only observed at doses above that required to fully inhibit IL-2 signaling [32].

Collectively, results from this investigation suggest that the reductions in PBNC in human RA patients may be an indirect consequence of the anti-inflammatory activity of CP-690,550 and/or the inhibition of JAK1 and JAK3 activity, but not JAK2. However, it is recognized that this compound may have additional pharmacological effects on either granulopoiesis and/or PBNC at exaggerated clinical dose levels where loss of selectivity and cross-inhibition of JAK2 may occur.

Macrophages and lymphocytes have a well established role in the onset and progression of arthritis, but the role of neutrophils has been less clear [33]. However, several reports looking at the role of neutrophils in both RA patients and in non-clinical models of inflammatory arthritis indicate that these cells are likely to be involved in both the onset and progression of arthritic disease, particularly in the process of joint degradation [26,28,33-42]. Neutrophils infiltrating into an arthritic joint can release proteolytic enzymes and reactive oxygen species which can increase inflammation and accelerate the destruction of the bone and cartilage [43-45]. Therefore, it is plausible based upon the present study that the inhibition of neutrophilia by CP-690,550, as observed in both human RA patients and in the rat AIA model, is a desirable and beneficial pharmacological effect of CP-690,550.

## Competing interests

All authors were full time employees of Pfizer Inc at the time this work was performed. This study was sponsored by Pfizer Inc.

## Authors' contributions

DMM participated in the design and coordination of the studies and writing the manuscript. MME, CLF, JDW, CJG JLH, MJS, and MLB carried out the *in vivo *and *in vitro *studies. MIJ carried out some of the *in vitro *assays and helped write the manuscript. XL helped coordinate the *in vitro *assays and editing of the manuscript. MED performed the PK analysis/modeling and writing of the manuscript. SKR helped design and coordinate the rat AIA bone marrow assays. NK contributed intellectually to the design the studies and editing of the manuscript. DLM participated in the design and coordination of the studies and helped to draft and edit the manuscript. All authors read and approved the final manuscript.
